# University of Louisville

**Published:** 1852-09

**Authors:** 


### THE WESTERN JOURNAL.

THIRD SERIES.—VOL. X.—NO. III.

LOUISVILLE, SEPTEMBER 1, 1852.

### UNIVERSITY OF LOUISVILLE.

It will be seen from the advertisement on the cover of our Journal, that the preliminary course of Lectures in the University of Louisville, will commence on the first Monday of October. We have remarked with satisfaction that the preliminary term in this school has been attended every year by growing numbers, and by this circumstance as well as others we have been inclined, more and more, to the extension of the regular term. Our belief is, that an announcement by all the schools to the effect that a change to five months had been made, would be well received by the profession; and we are convinced that it would be better to begin the course a month earlier in autumn than to continue it into the spring. We hope in a few years to see this step taken by all the schools. Whatever we may ultimately attain to, we doubt much if a term of six months would now be acceptable to students; but we think we cannot be mistaken in the opinion that they would generally approve of an extension of the term to five months. The Faculty of the University of Louisville regard the course given in October as virtually such an extension.

